# A comparative synteny analysis tool for target-gene SNP marker discovery: connecting genomics data to breeding in Solanaceae

**DOI:** 10.1093/database/bay047

**Published:** 2018-06-03

**Authors:** Junkyoung Choe, Ji-Eun Kim, Bong-Woo Lee, Jeong Hee Lee, Moon Nam, Youn-Il Park, Sung-Hwan Jo

**Affiliations:** 1 SEEDERS Inc, Daejeon 34015, Republic of Korea; 2 School of Medicine, Biological Sciences, Chungnam National University, Daejeon 34134, Republic of Korea

## Abstract

It is necessary for molecular breeders to overcome the difficulties in applying abundant genomic information to crop breeding. Candidate orthologs would be discovered more efficiently in less-studied crops if the information gained from studies of related crops were used. We developed a comparative analysis tool and web-based genome viewer to identify orthologous genes based synteny as well as sequence similarity between tomato, pepper and potato. The tool has a step-by-step interface with multiple viewing levels to support the easy and accurate exploration of functional orthologs. Furthermore, it provides access to single nucleotide-polymorphism markers from the massive genetic resource pool in order to accelerate the development of molecular markers for candidate orthologs in the Solanaceae. This tool provides a bridge between genome data and breeding by supporting effective marker development, data utilization and communication.

Database URL: http://tgsol.seeders.co.kr/scomp/

## Introduction

The explosion of genomic resources following advancements in sequencing and genotyping technologies has led to an increased need for data interpretation and opportunities for the development of new applications (1). The discovery of meaningful knowledge from the mass of available data has the potential to make a major impact on crop breeding programs. Comparative analysis is one of the applications that can connect genomic data to crop breeding. Using the information gained from comparative analyses among crops, target genes that regulate important agronomic traits can be directly used to develop molecular markers for crop improvement (2).

The Solanaceae include economically important crops such as tomato, potato, pepper and eggplant (3, 4). Since the reference genomes of potato, tomato and pepper were released (5–7), many follow-up studies and large-scale resequencing projects of major breeding lines have been performed. Currently, there is an imbalance in the quantity and quality of data among different Solanaceae species. The knowledge obtained from genetic studies of one crop may be efficiently applied to other less-studied, phylogenetically close species for the discovery of orthologs (1).

Several groups have developed web-based or standalone applications to facilitate comparisons among different species (8–12). For instance, the Sol Genomics Network (SGN) developed a comparative viewer that visualizes different types of maps and markers in Solanaceae species (13, 14). CrowsNest, another recently developed comparative genomics tool visualizes whole-genome alignments and synteny between monocot genomes (15). To become a more powerful tool for crop breeding, a comparative analysis viewer should include some additional functions that the previously developed applications lack, such as the abilities to easily search for orthologs of target genes and to quickly develop molecular markers.

We developed a web-based comparative synteny viewer to explore orthologous genes among tomato, pepper and potato, allowing searches for orthologs and markers. The tool also provides access to single nucleotide-polymorphism (SNP) markers from the massive genetic resource pool in order to accelerate the development of molecular markers of candidate orthologs. This tool will play an important role as a bridge between genome data and breeding programs by supporting the effective communication and utilization of data (available in the TGsol website; http://tgsol.seeders.co.kr/scomp/).

## Database construction and contents

Translational Genomics for Solanaceae (TGsol) database is the functional and customized web database providing valuable information from various genomic data to molecular breeding researchers. TGsol website is designed to work with most common browsers. No additional software is required. The ‘comparative synteny viewer’ pages in TGsol were written in HTML, Python and PHP code based on 10.0.29-MariaDB-0ubuntu0.16.04.1 Ubuntu 16.04 DATABASES and Code Igniter 3.1.2 PHP Platform. To explore orthologous gene among tomato, pepper and potato, sequence alignment tool such as BLAST (2.2.28+), CD-HIT (v. 4.6), ClustalW (v. 2.1) were used for comparing gene sequence similarity (16, 17, 18). Bioinformatics analysis software, such as BWA (v. 0.6.1-r104), SAMtools (v. 0.1.16) and several in-house parsing scripts (Python and Perl code) were used for identifying and constructing SNP marker database (19, 20). And we deposited source code in Source Forge web repository (https://sourceforge.net/projects/seederscs/) (current license status) and anyone can download this code scripts.

## Detection of homologs in Solanaceae

Solanaceae genomes are particularly amenable to comparative genomics analysis, because there have been relatively few genome rearrangements and duplications within the family, resulting in the same number of chromosomes (*n* = 12) and very similar gene content and order among the species, although the genome sizes vary somewhat (7). To detect candidate orthologous genes among the Solanaceae genomes, we collected gene sequences from several databases: tomato (*Solanum lycopersicum* Heinz 1706, SL2.40 and ITAG2.30) and potato [*Solanum tuberosum* Phureja DM1-3 516R44 (CIP801092) v4.3 and annotated by v3.4] from the SGN (https://solgenomics.net/), pepper (*Capsicum annuum* cv. CM334, v1.55) from the Pepper genome platform (http://peppergenome.snu.ac.kr/) and *Arabidopsis thaliana* from the Arabidopsis Information Resource (TAIR) (https://www.arabidopsis.org/) (Table 1). We grouped the homologous genes among those crops based on sequence similarity using the CD-HIT software with a sequence-identity cutoff of least 95%, which resulted in the prediction of a total of 17 635 homologous groups (17). Detailed information about the homologous genes is shown in Table 1.

**Table 1.** Summary of homologs based on sequence similarity among Solanaceae genomes

Crop	Number of genes	Number of homologous genes	Reference genome
Tomato	34 727	22 831 (65.7%)	ITAG 2.3
Pepper	34 915	18 554 (52.1%)	Pepper genome v1.5
Potato	39 028	34 369 (88.06%)	DM v4.3
Arabidopsis	35 386	12 715 (35.9%)	TAIR 10

## SNP marker pool from massive genome sequencing data

We detected the genome-wide SNPs from the massive genomic resources of tomato and pepper to develop genetic markers for candidate orthologous genes of *Solanaceae*. We collected whole-genome sequencing data for 234 tomato cultivars in the Short Reads Archive at the National Center for Biotechnology Information (NCBI, http://www.ncbi.nlm.nih.gov/Traces/sra/) (21). Information such as the individual code, cultivar, origin, identifier and sequence size (Gbp) of the data sets is provided on our website. We detected 598 993 raw SNPs among the 234 cultivars (22). To select more accurate and reliable SNPs for the development of molecular markers for genomic breeding, we chose 177 148 SNPs with minor allele frequencies >0.2. We also collected resequencing data from the F7∼9 recombinant inbred line (RIL), a cross between *C. annuum* Perennial and *C. annuum* Dempsey, available from the Pepper genome platform (http://peppergenome.snu.ac.kr/) (7). In total, we collected 8 702 453 SNPs detected in 120 resequencing data sets. Many potato lines are tetraploid, however, there is no accession in NCBI-SRA database a homozygous diploid line *S. tuberosum* Phureja, sequenced by Illumina Hiseq platform. So we have gather three RNA-seq data and identified 324 515 SNPs variation against reference potato genome (23). These SNP pool detected from tomato, pepper genomic data or potato RNAseq data were to database and can be applied to develop markers for target genes.

## Functionality of the synteny analysis tool

### Search for candidate orthologs of target genes

The identification of the loci of target genes is a key step in the development of molecular markers for crop breeding (24). In the Solanaceae, the abundant results of gene studies performed with tomato can be used to investigate target genes in other crops. For example, the number of published gene-level studies in the PubMed database is 8558 for tomato, 5326 for potato, and 1546 for pepper. Genetic information derived from heavily studied crops could be applied to less-studied crops through the identification of conserved homologous genes. In comparative genomics, orthologous genes are homologous genes evolved from a common ancestor and separated by a speciation event (25). Some orthologs have retained enough sequence similarity and physical colinearity along chromosomes to be identified as synteny blocks (26). The identification of orthologs and synteny blocks among closely related species can lead to the development of molecular markers for target genes, providing increased breeding efficiency (24).

**Figure 1.**
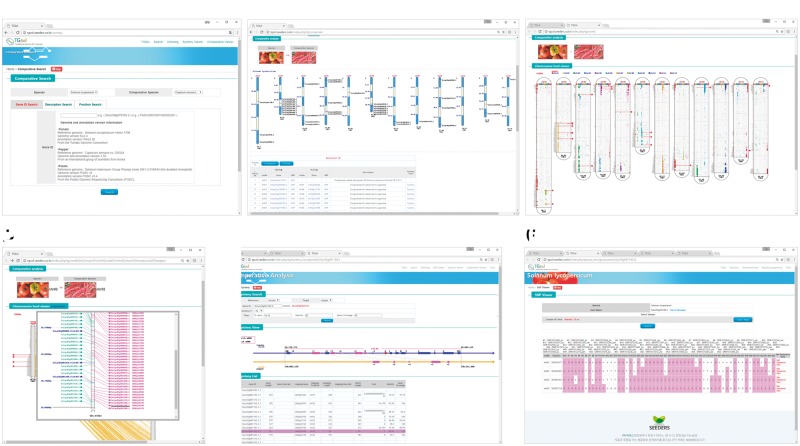
Interface of the comparative genomic viewer exploring orthologous genes between tomato and pepper genomes. (**A**) Search box for target genes. (**B**) Whole-genome viewer. (**C**) Chromosome comparison viewer (**D**) and more detailed viewer. (**E**) Synteny block viewer. (**F**) SNP database for development of markers for candidate orthologous genes.

We developed a graphical viewer with a user-friendly interface providing several levels of resolution to aid researchers in the exploration and identification of possible orthologous genes in Solanaceae crops. The interface provides a search box for a target gene before visualizing the comparison of two genomes (Figure 1A). The search box allows the user to search for a target gene in three different ways: by a gene identifier (gene ID), by a gene description or by the physical position in the tomato, pepper or potato genome. Gene ID numbers can be used to easily search for a gene using the ‘Gene ID search’ option. Since IDs of gene, transcript and proteins are different in case of potato gene annotation, we implemented enable to working well using any IDs of potato. If user does not have a particular gene ID, a partial or full gene description with corresponding target-gene functions can be used to search for candidate genes using the ‘Description search’ option. The user can narrow down the search by adding a physical position (chromosome number or target positions) of a target gene. If the user has the chromosomal position of a target gene from quantitative trait-loci mapping, e.g. the user can search for candidate genes by using the ‘Position search’ option.

Once the user has searched for a target gene or genes, the graphical viewer provides a table of results along with a simple image representing whole chromosomes with the overall distribution of homologous genes between two selected genomes (e.g. tomato and pepper; Figure 1B). The genes can be explored in more detail by clicking the links on the table to open the genome browser, SNP marker database or homologous clustering information or to zoom in using the chromosome comparison viewer (Figure 1C and D). With more progressive depth, the viewer can zoom in to show synteny blocks flanking a selected ortholog, providing the gene order, direction and description (Figure 1E).

## Viewer for orthologs of target genes

### (Level 1) whole-genome view

After searching for a target gene or genes, the comparative viewer starts at the whole-genome scale, providing information about homologous genes with a simple graphic image and table. In the example shown in Figure 1B, the two species being compared are shown in photo images at the top, with an ideogram of the 12 chromosomes of the target species showing the gene IDs and locations of the target genes below, and a table providing information about homologous genes at the bottom. From that page, the user can zoom in to view a part of the genome at higher resolution, change to a synteny block viewer, or open a genome browser to obtain available marker information about target or subject gene.

### (Level 2) chromosome comparison view

From the level 1 view, the user can zoom in to the chromosome comparison view (Figure 1C), which shows the overall distribution of homologous genes within all the chromosomes between two species (e.g. tomato and pepper). The chromosomes of the target genome are arranged in ellipse-shaped columns, with the chromosomes of the genomes being compared shown as vertical bars inside each ellipse. Colored blocks represent homologous genes identified based on sequence similarity between the two chromosomes. Genes selected as search targets by the user are shown as horizontal red lines. The chromosome comparison view illustrates the distribution of homologous genes between two species and can thus help to identify recombination break points for species differentiation.

Depending on the sequence similarity, the putative orthologs were identified between tomato and pepper, tomato and potato and pepper and potato, respectively. The chromosome comparison view can be zoomed in to a more detailed view showing candidate orthologous genes (Figure 1D). In the zoomed-in view, the user can see common genes identified in both species with homology on the same chromosome (pink color), homology on different chromosomes (navy color) and no homology (mint color) based on the significance cutoff. The user can click on the candidate homologous genes (pink color) to display detailed gene features and to move to the synteny viewer.

### (Level 3) synteny block view

The viewer can be zoomed in to explore syntenic blocks including a selected gene between two species. The synteny block viewer can provide critical evidence as to whether the target gene, neighboring genes and major homologs exist in a synteny block or in separate regions. The user can move the viewer window upstream and downstream and can zoom in and out to continuously explore regions of potential synteny. To more accurately identify orthologous genes, the viewer can apply a variety of syntenic quality parameters such as *e*-value, identity and sequence matching coverage.

An example of the synteny block viewer is given in Figure 1E. A selected gene is shown in the front-level viewer represented with the number ‘0’ and the red-colored box in the center of the displayed region. The displayed region can be changed by sliding the 0.2 Mbp window across the chromosome or by clicking the figure up to the number of genes to be included in the display. The filtering parameters can be adjusted based on the user’s purpose, and regions of high conservation can clearly be seen. The scope viewer can be used to identify orthologous genes.

## Link to the SNP database for marker development

From the table produced by the search for a target gene (Figure 1B), the user can access the SNP marker database directly by clicking the word ‘SNP’ beside the gene ID in the table. The SNPs detected in the target gene among the 234 tomato cultivars or 120 pepper RILs or potato RNAseq data are provided (Figure 1F). The user can adjust the SNP matrix by selecting samples and thus get a 1 kbp flanking sequence with each candidate SNP marker in txt format to copy directly. Another approach is to access the genome browser, which provides detailed gene information with the markers available within target genes. If the user clicks the gene ID in the table list, the genome browser opens a new window. Each feature in the genome browser provides functional annotation information with hyperlinks to the original web database page and various sequences (FASTA files). The SNP information for the target gene also provides flanking sequences that can be fed into primer design tool (i.e. Primer3 and others). The SNP marker database can be applied in practical use to develop markers for target genes.

### Future direction

This database was started as a part of efforts to make better use of genomic data for practical breeding efforts using comparative genomics tools. The step-by-step viewing interface of the database will support the exploration of candidate ortholog of multigene families or less conserved genes more easily by detecting homology and synteny. The production and accumulation of genomic data and the demand for applications of those complex data are still overflowing. We will therefore continue to add genomic data for more diverse accessions from NCBI and also develop molecular markers for future inclusion within the database. Those steady efforts to update and improve the database will help accelerate development of molecular marker for Solanaceae and other crops and lead to improved varieties with improved yield and quality, tolerance to unfavorable environmental conditions and resistance to disease.

## Funding

This work was supported by a grant from the Next-Generation BioGreen 21 Program (No. PJ01100301, PJ01313203), Rural Development Administration, Republic of Korea.

*Conflict of interest*. None declared.
